# Editorial: Dietary and nutrigenetic contributors to metabolic syndrome

**DOI:** 10.3389/fendo.2025.1632753

**Published:** 2025-06-18

**Authors:** Anoop Arunagiri, Maroof Alam, Erez Dror, Melkam A. Kebede

**Affiliations:** ^1^ Department of Biological Sciences, East Tennessee State University, Johnson City, TN, United States; ^2^ Department of Internal Medicine, University of Michigan Medical School, Ann Arbor, MI, United States; ^3^ Department of Developmental Biology and Cancer Research, Institute for Medical Research Israel-Canada, Faculty of Medicine, The Hebrew University of Jerusalem, Jerusalem, Israel; ^4^ Charles Perkins Centre, The University of Sydney, Sydney, Australia, and School of Medical Sciences, Faculty of Medicine and Health, The University of Sydney, Sydney, NSW, Australia

**Keywords:** diet, metabolism, obesity, metabolic syndrome, public health

This editorial introduces a Research Topic exploring the diverse relationships between diet, metabolism, and health. The collection of eight studies examines topics ranging from dietary trends and obesity to the impact of specific nutrients and ultra-processed foods on metabolic health and quality of life. Investigations into the effects of low-carbohydrate diets on metabolic syndrome, the link between thyroid hormone sensitivity and insulin resistance, and the prevalence of anemia in pregnant women are presented. Furthermore, the relationship between dietary nitrates/nitrites and gut metabolites in metabolic syndrome, and the weight-loss-independent effects of fasting on human growth hormone are highlighted. Collectively, these studies underscore the multifaceted nature of nutritional science and offer valuable insights for future research and public health strategies aimed at improving health outcomes.

The complex connection between diet, metabolism, and human health continues to be a central focus of scientific inquiry. This Research Topic brings together a collection of eight distinct studies, each shedding light on specific aspects of this complex interplay across diverse populations and physiological contexts. From examining dietary trends and their association with obesity to investigating the nuanced effects of different fatty acids and the impact of ultra-processed foods on quality of life, these articles collectively highlight the elaborate nature of nutritional science and its profound influence on well-being.

Alhusseini et al., in the article “*Dietary Trends and Obesity in Saudi Arabia*,” provide valuable insights into the eating habits of a cohort of Saudi adults. The study highlights a significant prevalence of non-specific dietary patterns alongside the adoption of popular diets like intermittent fasting and ketogenic diets, primarily for weight loss. Notably, the research identifies key demographic and lifestyle factors, such as age, region, exercise, and meal timing, as predictors of dietary plan adherence and reveals a significant association between BMI and the type of diet followed. This work emphasizes the ongoing challenge of obesity in the region and the crucial need for increased awareness and sustainable, balanced nutrition.

Moving to the cellular and molecular level, Liu et al., in “*Comparison of the effects of monounsaturated fatty acids and polyunsaturated fatty acids on the lipotoxicity of islets*,” delve into the protective roles of different unsaturated fatty acids against saturated fatty acid-induced damage in pancreatic islets. Through *in vitro* and *in vivo* experiments, the study reveals that both MUFAs and PUFAs can mitigate lipotoxicity, with oleic acid showing superior effects in reducing ER stress in cells and olive oil demonstrating notable benefits in enhancing insulin sensitivity in a mouse model. These findings suggest a promising avenue for nutritional interventions in managing metabolic diseases like type 2 diabetes.

Shifting focus to the impact of modern dietary patterns, “*Ultra-processed food consumption and quality of life: a cross-sectional study in Iranian women*,” by Hosseininasab et al. explore the association between the intake of ultra-processed foods and quality of life in overweight and obese Iranian women. The study reveals a significant inverse relationship, with higher UPF consumption linked to poorer scores in physical role, vitality, and mental health domains, even after adjusting for key confounders. This research underscores the potential detrimental effects of high UPF diets on overall well-being and advocates for limiting their intake to improve quality of life.

Abdulghani et al., in “The Effect of a Nurse-led Low Carbohydrate Regimen on Anthropometric and Laboratory Parameters of Patients with Metabolic Syndrome: A Quasi-Experimental Study,” investigate the impact of a structured dietary intervention on individuals with metabolic syndrome in Iraq. The three-month nurse-led low-carbohydrate regimen resulted in significant improvements in various anthropometric measures, including BMI and waist circumference, as well as key laboratory parameters such as total cholesterol, triglycerides, HDL, FBS, and HbA1c. This study highlights the potential of personalized dietary interventions in managing metabolic syndrome and improving cardiometabolic health markers.

Exploring the subtle hormonal regulation of metabolism, Wei et al., in the article “*Associations between Sensitivity to Thyroid Hormones and Insulin Resistance in Euthyroid Adults with Obesity*,” examine the relationship between central thyroid hormone sensitivity indices and insulin resistance in a cohort of euthyroid adults with obesity in China. The study reveals a significant association between reduced central thyroid hormone sensitivity and increased adipose tissue insulin resistance, even after adjusting for multiple risk factors. This research contributes to our understanding of the detailed interplay between thyroid function and insulin sensitivity in the context of obesity and metabolic dysfunction.

Addressing a critical health issue in a specific population, Nasir et al., in “*Magnitude of Anemia and associated factors among pregnant women attending Antenatal Care in governmental health facilities of Shashemene Town, Oromia region, Ethiopia*,” assess the prevalence and risk factors associated with anemia in pregnant women. The study reports a substantial anemia prevalence of 30.9% and identifies key factors such as low dietary diversity, lack of deworming, young age, malnourishment, no contraceptive use, and lack of iron supplementation as significantly associated with increased risk. These findings underscore the need for targeted interventions to improve maternal health outcomes in this region.

Mirzababaei et al., in “The interaction between dietary Nitrates/Nitrites intake and Gut Microbial Metabolites on Metabolic syndrome: A cross-sectional study,” investigate the complex interplay between dietary nitrate/nitrite intake from different sources (animal vs. plant) and gut microbial metabolites (TMAO and KYN) on metabolic syndrome in Iranian adults. The study reveals differential associations, with higher intake of nitrates/nitrites from animal sources combined with high TMAO and KYN levels being linked to increased risk of some MetS components, while plant-based sources showed some protective effects. This research highlights the importance of considering both dietary sources and gut microbiome interactions in understanding MetS risk.

Finally, Horne et al., in “Weight Loss-Independent Changes in Human Growth Hormone During Water-Only Fasting: A Secondary Evaluation of a Randomized Controlled Trial,” delve into the metabolic effects of short-term fasting. The study demonstrates that a 24-hour water-only fast increases human growth hormone (HGH) levels independently of weight loss. Furthermore, it suggests that individuals with lower baseline HGH experience a more pronounced relative increase and a trend towards greater improvement in insulin resistance. This research indicates that the benefits of short-term fasting may extend beyond weight reduction and involve significant hormonal changes.

Collectively, the diverse studies within this Research Topic offer a comprehensive exploration of the multifaceted relationships between diet, metabolism, and health across various populations and physiological states. From identifying dietary patterns and their associations with obesity to elucidating the cellular mechanisms of nutrient action and the hormonal responses to dietary interventions, these articles contribute valuable insights that can inform future research, dietary guidelines, and public health strategies aimed at improving overall well-being.

